# Comparison of epidemiological features, clinicopathological features, and treatments between premenopausal and postmenopausal female breast cancer patients in western China: a retrospective multicenter study of 15,389 female patients

**DOI:** 10.1002/cam4.1503

**Published:** 2018-04-19

**Authors:** Fan Feng, Yuxian Wei, Ke Zheng, Yujing Li, Lu Zhang, Tielin Wang, Yanli Zhang, Hongyuan Li, Guosheng Ren, Fan Li

**Affiliations:** ^1^ Department of Thyroid and Breast Surgery The First Affiliated Hospital of ChongQing Medical University Friendship Road Yu‐Zhong District, ChongQing 400016 China; ^2^ Department of Oncology The First Affiliated Hospital of ChongQing Medical University Friendship Road Yu‐Zhong District, ChongQing 400016 China

**Keywords:** Breast cancer, hormone receptor, postmenopausal, pregnancy, premenopausal

## Abstract

Premenopausal and postmenopausal breast cancers are considered different types. Thus, this study aimed to explore differences in risk factors, epidemiological features, clinicopathological features, and treatment modes of premenopausal breast cancer compared to postmenopausal patients in western China. This was a hospital‐based, retrospective, multicenter epidemiological study of patients with breast cancer. Using the Western China Clinical Cooperation Group database, we obtained the records of 15,389 female breast cancers between January 2010 and April 2017. These patients were divided into premenopausal and postmenopausal groups, and their risk factors, epidemiological feature, clinicopathological features, and treatment modes were compared. Chi‐square tests, *t*‐test, and the multivariate logistic regression analysis were applied for statistical analysis. A total of 8395 patients were categorized as premenopausal, and 6994 patients were categorized as postmenopausal. Their risk factors, epidemiological features, clinicopathological features, and treatment modes were compared. Premenopausal patients with breast cancer had a greater tumor diameter at diagnosis (*P* = 0.008); higher rates of estrogen receptor (ER) expression (*P* < 0.0001), progesterone receptor (PR) expression (*P* < 0.0001), negative human epidermal growth factor receptor 2 (HER2) expression (*P* = 0.015), and negative P53 expression (*P* < 0.0001); and higher proportions of receiving breast‐conserving surgery and breast reconstruction (*P* < 0.0001), chemotherapy (*P* < 0.0001), radiotherapy (*P* < 0.0001), and endocrine therapy (*P* < 0.0001). The ethnicity, age at menarche, marital status, number of pregnancies, and number of births were the risk factors for age at diagnosis of breast cancer before or after menopause in western China. We found that the fall in the fertility rate, early menarche age, married, and less breastfeeding might have increased the possibility of premenopausal breast cancer. Significant differences exist in the tumor size, hormone receptor state, HER2 expression, epidemiological features, and treatment modes between premenopausal and postmenopausal female breast cancer patients in western China. Its further implementation requires prospective clinical testing.

## Introduction

Breast cancer is the most common cancer in women, and its incidence is increasing annually worldwide 1, 2. Breast cancer is also a heterogeneous disease, divisible into various clinical subtypes, and the pathogenesis is not clear 3. As early as the 1970s, De Waard proposed the concept that breast cancers develop by two distinct pathways, each with a different age‐specific incidence rate curve 4, 5. The first pathway results in mainly premenopausal tumors with peak occurrence early in life. The second pathway results in predominantly postmenopausal cancers with peak incidence later in life, similar to late‐onset estrogen receptor(ER)‐positive cancers.

In China, the data from both Shanghai and Beijing showed two breast cancer age peaks, one at 45–55 years and the other at 70–74 years 6. The mean age at diagnosis of breast cancer in China is 45–55 years, which is considerably younger than that for western women, with 57.4% of women diagnosed before the age of 50 years and 62.9% of women diagnosed while still premenopausal; the peak incidence occurred after menopause in developed countries 7. This result suggests the possibility that certain differences in the pathogenesis of breast cancer may exist between Chinese women and women in Western populations.

Few studies have investigated the risk factors that may influence the age at diagnosis of breast cancer. The difference in the clinicopathologic features and treatment modes between premenopausal and postmenopausal patients with breast cancer is not known. Some scholars have predicted that the incidence of hormone receptor‐positive breast cancer is affected by the menopause transition 8, 9. Nonetheless, and somewhat paradoxically, it has been reported that menopause significantly affects the incidence of ER‐negative breast cancer but not that of ER‐positive breast cancer 10, 11. Presently, this issue is controversial.

Currently, no study has compared the difference between premenopausal and postmenopausal breast cancers in China. Therefore, the main objective of this study was to assess the epidemiological characteristics, clinicopathologic features, and treatment modes between premenopausal and postmenopausal breast cancers to make treatment decisions and improve patient prognoses, as well as to provide valuable insights into what may influence the age at diagnosis of breast cancer in western China.

## Materials and Methods

### Study design

The Western China Clinical Cooperation Group (WCCCG) was established in 2008 and includes 23 breast cancer centers in nine provinces in western China. A total of 18,000 patients with breast cancer are included in the database. Male patients and female patients without menopausal status and age at diagnosis were excluded from the study. In total, 15,389 patients with breast cancer who were diagnosed between 1 January 2009 and 30 April 2017 were included in the retrospective multicenter database analysis. Among them, 8395 patients (54.55%) were divided into premenopausal group, and 6994 patients (45.45%) were divided into postmenopausal group. This observational study was based entirely on data extracted from patient medical records and was approved by the ethics committee of each participating center.

### Patients

The data for this study, including demographic data and tumor data, were extracted from the medical records of the patients included herein by trained data collectors at each center and were analyzed anonymously.

### Data collection

Demographic data included information regarding the age at diagnosis, race, age at menarche, marital status, and number of pregnancies, number of births, breastfeeding history, and body mass index. Clinical characteristics consisted of tumor laterality, location in the breast, axillary and supraclavicular lymph node status, and size in cm. The tumors were classified according to initial disease symptoms and signs and whether there was distant metastasis in the body. The following pathologic characteristics were evaluated in the study: tumor histological types, axillary lymph node metastases, numbers of positive axillary lymph nodes, the presence of lymph vascular invasion, tumor grade, ER and PR status, HER2 and P53 expression, and Ki67 status. Data regarding treatments were also collected and included the chemotherapy regimens, radiotherapy regimens, anti‐HER2 therapy regimens, endocrine therapy regimens, types of surgeries, and axillary lymph node dissection procedures.

### Pathological grading and staging criteria

The tumor was graded according to the Bloom–Richardson classification (Nottingham grading) 12. Staging of breast cancer size was performed according to the American Joint Committee on Cancer (AJCC) TNM staging system (from 1997 and 2002) 13. All centers use the same criteria.

### Statistical analyses

Statistical analyses were performed using the Statistical Package for the Social Sciences, version 22.0 (SPSS Inc., Chicago, IL, USA). The differences in the demographic, clinical, and pathological characteristics and in treatments between the two groups were analyzed using Student's *t*‐test in the case of quantitative variables and chi‐square tests and Fisher's exact test in the case of categorical variables. Multivariate logistic regression analyses were performed to assess the associations between menopausal status and several variables, and odds ratios (ORs) were calculated based on 95% confidence intervals. Variables with univariate results of *P* < 0.05 were included in the multivariate model. All statistical tests were considered significant when *P* < 0.05.

## Results

### Comparison of the baseline characteristics

The comparison of the baseline characteristics of premenopausal and postmenopausal groups is summarized in Table 1. The mean ages at diagnosis of the patients in premenopausal and postmenopausal groups were 42.8 and 58.2 years, respectively, and there was a difference in the distributions of the ages at which patients were diagnosed between the two groups (*P* < 0.0001). The proportion of patients of Han ethnicity was lower in premenopausal group than in postmenopausal group (94.69% vs. 96.61%, respectively, *P* < 0.0001). Most of the patients in the two groups experienced menarche at 13–14 years [*n* = 4396 (52.36%) and *n* = 3456 (49.41%), respectively]. Patients in premenopausal group were younger at the time of menarche (younger than 12 years) than patients in postmenopausal group. In addition, more patients in postmenopausal group experienced menarche at an age older than 15 years (*P* < 0.0001).We also observed differences in the marital status (*P* < 0.0001), number of pregnancies (*P* < 0.0001), number of births (*P* = 0.002), and breastfeeding history (*P* < 0.0001) between the two groups. The mean body mass indexes (BMI) of the patients in premenopausal and postmenopausal groups were 23.1 and 23.6, respectively. An analysis of BMI showed that premenopausal group included more patients who had a BMI <25.0 compared with that postmenopausal group [*n* = 3693 (43.99%) and *n* = 2825 (40.39%), respectively] (*P* < 0.0001).

**Table 1:** Baseline characteristics of the patients with breast cancer

Characteristics	Total (*N* = 15,389)		Premenopausal (*N* = 8395)		Postmenopausal (*N* = 6994)		*P* value
	*N*	%	*N*	%	*N*	%	
Age							
Mean ± SD	49.6 ± 11.1		42.8 ± 7.0		58.2 ± 8.5		<0.0001^a^
Race/ethnicity							
Han	14,706	95.51	7949	94.69	6757	96.61	<0.0001^b^
Other	683	4.44	446	5.31	237	3.39	
Age at menarche (years)							
≤10	30	0.19	20	0.24	10	0.14	<0.0001^b^
11–12	1844	11.98	1191	14.19	653	9.34	
13–14	7852	50.99	4396	52.36	3456	49.41	
15–16	3981	25.85	2089	24.88	1892	27.05	
17–18	1349	8.76	583	6.94	766	10.95	
≥19	284	1.84	98	1.17	186	2.66	
Missing data	49	0.32	18	0.21	31	0.44	
Marital status							
Married	14,960	97.16	8188	97.53	6772	96.83	<0.0001^c^
Never married	138	0.90	110	1.31	28	0.40	
Widowed/divorced	276	1.79	89	1.06	187	2.67	
Missing data	15	0.10	8	0.10	7	0.10	
Number of pregnancies							
0	3239	21.04	1790	21.32	1449	20.72	<0.0001^b^
1	3848	24.99	2216	26.40	1632	23.33	
2	3228	20.96	1807	21.52	1421	20.32	
3	2228	14.47	1230	14.65	998	14.27	
4	1408	9.14	715	8.52	693	9.91	
≥5	1413	9.18	623	7.42	790	11.30	
Missing data	25	0.16	14	0.17	11	0.16	
Number of births							
0	2753	17.88	1584	18.87	1169	16.71	<0.0001^b^
1	7086	46.02	4404	52.46	2682	38.35	
2	3579	23.24	1887	22.48	1692	24.19	
3	1198	7.78	378	4.50	820	11.72	
4	468	3.04	97	1.16	371	5.30	
≥5	282	1.83	32	0.38	250	3.57	
Missing data	23	0.15	13	0.15	10	0.14	
Breastfeeding history							
No	1543	10.02	900	10.72	643	9.19	0.002^b^
Yes	5050	32.80	2685	31.98	2365	33.81	
Missing data	8796	57.12	4810	57.30	3986	56.99	
BMI							<0.0001^a^
Mean ± SD	23.3 ± 2.5		23.1 ± 2.4		23.6 ± 2.7		<0.0001^b^
<18.5	442	2.87	252	3.00	190	2.72	
18.5~24.9	6076	39.46	3441	40.99	2635	37.68	
25.0~29.9	2184	14.18	983	11.71	1201	17.17	
≥30.0	308	2.00	129	1.54	179	2.56	
Missing data	6379	41.43	3590	42.76	2789	39.88	

a Student's *t*‐test.

b Chi‐square test.

c Fisher's exact test.

### Comparison of clinical characteristics

Table 2 shows significant differences in the occurrence of breast pain (8.33% vs. 9.75%, *P* = 0.002), nipple discharge (2.30% vs. 2.83%, *P* = 0.037), and nipple inversion (1.21% vs. 2.04%, *P* < 0.0001). Patients in postmenopausal group were more likely to have a positive axillary lymph node status (29.40% vs. 27.95%, *P* = 0.031) and supraclavicular lymph node status (5.85% vs. 4.93%, *P* = 0.007). However, no significant difference in the incidence of distant metastases was observed between the two groups (1.70% vs. 2.10%, *P* = 0.073). Regarding the tumor size, most patients in the two groups had a tumor size between 2 and 5 cm, whereas significantly more patients in premenopausal group had large tumors (8.1% vs. 7.55%, *P* = 0.008).

**Table 2:** Clinical characteristics of the tumors

Characteristics	Total (*N* = 15,389)		Premenopausal (*N* = 8395)		Postmenopausal (*N* = 6994)		*P* value
	*N*	%	*N*	%	*N*	%	
Breast lump							
Yes	14,485	94.07	7913	94.26	6572	93.97	0.482^a^
No	904	5.87	483	5.75	421	6.02	
Breast pain							
Yes	1381	8.97	699	8.33	682	9.75	0.002^a^
No	14,008	90.97	7697	91.69	6311	90.23	
Nipple discharge							
Yes	391	2.54	193	2.30	198	2.83	0.037^a^
No	14,998	97.40	8203	97.71	6795	97.15	
Nipple inversion							
Yes	254	1.65	111	1.32	143	2.04	<0.0001^a^
No	15,135	98.29	8285	98.69	6850	97.94	
Tumor location in breast							
3 o'clock	385	2.50	214	2.55	171	2.44	<0.0001^a^
6 o'clock	259	1.68	138	1.64	121	1.73	
9 o'clock	475	3.08	254	3.03	221	3.16	
12 o'clock	813	5.28	478	5.69	335	4.79	
Upper inner quadrant	1975	12.83	1190	14.18	785	11.22	
Lower inner quadrant	577	3.75	326	3.88	251	3.59	
Upper outer quadrant	5637	36.61	3238	38.57	2399	34.30	
Lower outer quadrant	1119	7.27	624	7.43	495	7.08	
Nipple–areola	910	5.91	449	5.35	461	6.59	
Missing data	3239	21.04	1484	17.68	1755	25.09	
Axillary lymph lode status							
Positive	4402	28.59	2346	27.95	2056	29.40	0.031^b^
Negative	10,097	65.57	5578	66.44	4519	64.61	
Missing data	890	5.78	471	5.61	419	5.99	
Supraclavicular lymph lode status							
Positive	823	5.34	414	4.93	409	5.85	0.007^b^
Negative	13,557	88.04	7480	89.10	6077	86.89	
Missing data	1009	6.55	501	5.97	508	7.26	
Tumor size (cm)							
≤1	820	5.33	485	5.78	335	4.79	0.008^a^
>1,≤2	4456	28.94	2370	28.23	2086	29.83	
>2,≤5	7158	46.49	3907	46.54	3251	46.48	
>5	1208	7.85	680	8.10	528	7.55	
Missing data	1747	11.35	953	11.35	794	11.35	
Distant metastasis							
Positive	290	1.88	143	1.70	147	2.10	0.073^a^
Negative	14,951	97.10	8165	97.26	6786	97.03	
Missing data	148	0.96	87	1.04	61	0.87	

a Chi‐square test.

b Fisher's exact test.

### Comparison of pathological characteristics

Pathological characteristics are displayed in Table 3. Regarding ER, PR, human epidermal growth factor receptor‐2 (HER‐2), P53 status and Ki67, with which the patients receiving immunohistochemistry testing presented, we found that patients in premenopausal group were more likely to show positive expression of ER (59.14% vs. 54.86%, *P* < 0.0001) and PR (55.76% vs. 43.78%, *P* < 0.0001) than postmenopausal group. Premenopausal patients also had higher proportions of double‐positive expression of ER and PR (i.e., ER+/PR+; 50.46% vs. 40.43%, *P* < 0.0001) and single‐positive expression of PR (i.e., ER−/PR+; 5.28% vs. 3.33%, *P* < 0.0001). Conversely, patients in postmenopausal group presented with double‐negative expression of ER and PR (i.e., ER−/PR−; 35.72% vs. 31.40%, *P* < 0.0001) and single‐positive expression of ER (i.e., ER+/PR−; 14.20% vs. 8.29%, *P* < 0.0001) more frequently. Moreover, the proportion of patients with a positive HER2 status (14.46% vs. 13.48%, *P* = 0.015) and P53 status (29.47% vs. 25.63%, *P* < 0.0001) was higher in postmenopausal group. However, no significant difference in the incidence of triple‐negative or Ki67 was observed between the two groups.

**Table 3:** Pathological characteristics of the tumors

Characteristics	Total (*N* = 15,389)		Premenopausal (*N* = 8395)		Postmenopausal (*N* = 6994)		*P* value
	*N*	%	*N*	%	*N*	%	
Tumor histology							
Carcinoma in situ	801	5.20	413	4.92	388	5.55	0.053^a^
Invasive carcinoma	14,008	90.97	7712	91.86	6296	90.02	
Missing data	580	3.77	270	3.22	310	4.43	
Axillary lymph nodes metastasis							
Yes	6584	42.76	3612	43.03	2972	42.49	0.914^a^
No	7210	46.82	3962	47.19	3248	46.44	
Missing data	1595	10.36	821	9.78	774	11.07	
No. of positive axillary lymph nodes							
0 (N0)	7210	46.82	3962	47.19	3248	46.44	0.068^a^
1–3 (N1)	4125	26.79	2285	27.22	1840	26.31	
4–9 (N2)	1572	10.21	877	10.45	695	9.94	
≥10 (N3)	887	5.76	450	5.36	437	6.25	
Missing data	1595	10.36	821	9.78	774	11.07	
Lymphovascular invasion							
Yes	270	1.75	155	1.85	115	1.64	0.099^a^
No	6873	44.64	3594	42.81	3279	46.88	
Missing data	8246	53.55	4646	55.34	3600	51.47	
Tumor grade							
I	554	3.60	292	3.48	262	3.75	0.588^a^
II	5384	34.97	2776	33.07	2608	37.29	
III	1893	12.29	1002	11.94	891	12.74	
Uncertain	1546	10.04	785	9.35	761	10.88	
Missing data	5988	38.89	3527	42.01	2461	35.19	
ER+/PR+							
Yes	7269	50.46	4236	40.43	2828	41.25	<0.0001^a^
No	7324	45.21	3795	53.35	3731	52.56	
Missing data	796	4.34	364	6.22	435	6.19	
ER+/PR−							
Yes	1722	8.29	696	14.20	993	14.41	<0.0001^a^
No	12,871	87.21	7321	79.40	5553	79.40	
Missing data	796	4.50	378	6.41	448	6.19	
ER−/PR+							
Yes	1722	5.28	443	3.33	233	14.41	<0.0001^a^
No	12,871	90.22	7574	90.26	6313	79.40	
Missing data	796	4.50	378	6.41	448	6.19	
ER−/PR−							
Yes	4916	31.40	2636	35.72	2498	34.69	<0.0001^a^
No	9677	64.26	5395	58.06	4061	59.12	
Missing data	796	4.34	364	6.22	435	6.19	
HER2 status							
Yes	2143	13.92	1132	13.48	1011	14.46	0.015^a^
No	8063	52.36	4496	53.56	3567	51.00	
Uncertain	2475	16.07	1257	14.97	1218	17.41	
Missing data	2708	17.59	1510	17.99	1198	17.13	
Triple‐negative							
Yes	2372	15.40	1279	15.24	1093	15.63	0.502^a^
No	13,017	84.54	7116	84.76	5901	84.37	
P53 status							
Yes	4213	27.36	2152	25.63	2061	29.47	<0.0001^a^
No	2304	14.96	1060	12.63	1244	17.79	
Missing data	8872	57.62	5183	61.74	3689	52.75	
Ki67							
≥14	4772	30.99	2534	30.18	2238	32.00	0.715^a^
14	2712	17.61	1452	17.30	1260	18.02	
Missing data	7905	51.34	4409	52.52	3496	49.99	
ER status							
Yes	8802	59.14	4965	54.86	3837	29.47	<0.0001^a^
No	5816	36.70	3081	39.10	2735	17.79	
Missing data	771	4.16	349	6.03	422	52.75	
PR status							
Yes	4772	55.76	4681	43.78	3062	32.00	<0.0001^a^
No	2712	39.93	3352	50.03	3499	18.02	
Missing data	7905	4.31	362	6.19	433	49.99	

a Chi‐square test.

### Comparison of treatment modes

We found that most patients received chemotherapy and that patients in premenopausal group received chemotherapy (85.06% vs. 75.72%, *P* < 0.0001), radiotherapy (20.31% vs. 13.14%, *P* < 0.0001), and endocrine therapy (29.77% vs. 22.79%, *P* < 0.0001) more frequently. Of the 15,389 patients included in the study, 14,521 patients underwent surgery. Patients in premenopausal group were more likely to undergo breast‐conserving surgery, simple mastectomy, and breast reconstruction and were less likely to undergo modified radical mastectomy than patients in postmenopausal group (*P* < 0.0001). The comparison of the treatment characteristics between the two groups is presented in Table 4.

**Table 4:** Treatments of the patients with breast cancer

Characteristics	Total (*N* = 15,389)		Premenopausal (*N* = 8395)		Postmenopausal (*N* = 6994)		*P* value
	*N*	%	*N*	%	*N*	%	
Chemotherapy							
Yes	12,437	80.77	7141	85.06	5296	75.72	<0.0001^a^
No	2294	14.90	960	11.44	1334	19.07	
Missing data	668	4.34	294	3.50	374	5.35	
Radiotherapy							
Yes	2624	17.04	1705	20.31	919	13.14	<0.0001^a^
No	12,058	78.31	6373	75.91	5685	81.28	
Missing data	707	4.59	317	3.78	390	5.58	
Anti‐HER2 therapy							
Yes	136	0.88	75	0.89	61	0.87	0.978^a^
No	14,571	94.63	8018	95.51	6553	93.69	
Missing data	682	4.43	302	3.60	380	5.43	
Endocrine therapy							
Yes	4093	26.58	2499	29.77	1594	22.79	<0.0001^a^
No	10,584	68.74	5574	66.40	5010	71.63	
Missing data	712	4.62	322	3.84	390	5.58	
Type of surgery							
Modified radical mastectomy	11,467	74.47	6061	72.20	5406	77.29	<0.0001^a^
Breast‐conserving surgery	1582	10.27	986	11.75	596	8.52	
Simple mastectomy	793	5.15	451	5.37	342	4.89	
Radical mastectomy	196	1.27	115	1.37	81	1.16	
Extensive radical mastectomy	103	0.67	58	0.69	45	0.64	
Breast reconstruction	380	2.47	314	3.74	66	0.94	
Missing data	868	5.64	410	4.88	458	6.55	
Axillary lymph node dissection							
Yes	13,065	84.85	7145	85.11	5920	84.64	0.058^a^
No	1575	10.23	901	10.73	674	9.64	
Missing data	749	4.86	349	4.16	400	5.72	
Level of axillary lymph node dissection							
Level I, II	9504	61.72	5220	62.18	4284	61.25	0.485^a^
Level III	1867	12.12	1009	12.02	858	12.27	
Missing data	4018	26.09	2166	25.80	1852	26.48	

a Chi‐square test.

### Multivariate logistic regression analysis of premenopausal breast cancer‐related risk factors among all breast cancer patients

Multivariate logistic regression analysis indicated following risk factors were related to premenopausal breast cancer: ethnicity, age at menarche, marital status, number of pregnancies, and number of births. Compared with referent (Han; age at menarche ≤10; married; absence of a history of pregnancy and birth): (1) other ethnicities and number of pregnancy ≥1 were associated with elevated premenopausal breast cancer possibility (OR > 1, *P* < 0.05); (2) and increase in age at menarche, never married, widowed/divorced, and number of birth ≥1 were associated with decreased premenopausal breast cancer possibility (OR<1, *P* < 0.05) among all breast cancer patients. All the results of multivariate logistic regression analysis are listed in Table 5.

**Table 5:** Multivariate logistic regression analysis of premenopausal breast cancer‐related risk factors

Factors	B	S_b_	*χ* ^2^	*P* value	OR (95% CI)
Ethnicity					
Han^a^					
Other	0.87	0.091	91.048	<0.001	2.388 (1.997–2.855)
Age at menarche					
≤10^a^					
11–12	−1.038	0.413	6.33	0.012	0.354 (0.158–0.795)
13–14	−1.013	0.141	51.852	<0.001	0.363 (0.276–0.479)
15–16	−0.71	0.133	28.356	<0.001	0.492 (0.378–0.638)
17–18	−0.6	0.135	19.673	<0.001	0.549 (0.421–0.716)
≥19	−0.295	0.143	4.243	0.039	0.745 (0.563–0.986)
Marital status					
Married^a^					
Never married	−0.88	0.138	40.648	<0.001	0.415 (0.317–0.544)
Widowed/ divorced	−1.832	0.256	51.151	<0.001	0.16 (0.097–0.264)
Number of pregnancies					
0^a^					
1	0.49	0.095	26.637	<0.001	1.632 (1.355–1.965)
2	0.668	0.081	67.892	<0.001	1.95 (1.664–2.286)
3	0.359	0.078	21.342	<0.001	1.432 (1.229–1.667)
Number of births					
0^a^					
1	−2.895	0.214	183.38	<0.001	0.055 (0.036–0.084)
2	−3.071	0.205	225.333	<0.001	0.046 (0.031–0.069)
3	−2.496	0.204	149.986	<0.001	0.082 (0.055–0.123)
4	−1.48	0.208	50.452	<0.001	0.228 (0.151–0.343)
≥5	−0.835	0.228	13.392	<0.001	0.434 (0.278–0.679)

a Referent.

## Discussion

Overall, it is of great interest that a significant difference exists between premenopausal and postmenopausal female breast cancer patients and we found some factors that are associated with elevated or decreased premenopausal breast cancer possibility. The related research is rare.

Consistent with the results of other scholars in China 6, 14, the median age at diagnosis was 49.6 years for all breast cancer patients in this study. The premenopausal patients accounted for 54.55% of the total breast cancer cases, the proportion was significantly higher than that of postmenopausal patients, and that differed from the corresponding proportion in a Western population 15. This finding suggests the possibility that certain differences in the pathogenesis of breast cancer, considered to be related to ethnicity, age at menarche, marital status, number of pregnancies, number of births, and breastfeeding history 16, 17, 18, 19, may exist between premenopausal and postmenopausal patients.

The onset of the premenopausal peak is considered related to a birth cohort effect, resulting from changes in the menstrual and reproductive patterns and other lifestyle changes 20, 21. Further research results in our study supported this possibility. First, multivariate logistic regression analysis indicated that other ethnicities and number of pregnancy ≥1 were associated with elevated premenopausal breast cancer possibility (OR > 1, *P* < 0.05). We found a significantly higher proportion of the minority nationalities and times of pregnancy ≥1 in premenopausal women. It was reported that the incidence age of breast cancer of minority nationalities was earlier than that of the Han nationality 22. Second, multivariate logistic regression analysis showed that increase in age at menarche, never married, widowed/divorced, and number of birth ≥1 were associated with decreased premenopausal breast cancer possibility (OR < 1, *P* < 0.05). We found that the age of postmenopausal women experienced menarche was older than that of premenopausal women in this study. Previous studies have suggested that early age at menarche is a risk factor leading to the advanced onset of breast cancer 16, 23, 24. We also noted that a higher proportion of postmenopausal women had more than two births and a history of breastfeeding. A meta‐analysis of 47 studies in 30 countries showed that breastfeeding could reduce the risk of breast cancer 25. Bao concluded that increased numbers of births per woman were associated with a reduced risk of breast cancer for postmenopausal women 17. We found the number of pregnancies was positively associated with risk of premenopausal breast cancer, but increased number of births decreased the risk of that. Because of the one‐child policy and some other reasons, Chinese women may not to give a birth after pregnancy. Here come to a conclusion that only pregnancy but no childbirth might increase the risk of premenopausal breast cancer. We speculate that the fall in the fertility rate 26, 27, early menarche age, the married and less breastfeeding might have increased the possibility of premenopausal breast cancer.

A Westernized lifestyle, particularly an increase in the obesity prevalence and physical inactivity in recent decades, is likely to affect the observed rise in breast cancer incidence 28, 29. Obesity was considered a mechanism of breast cancer in postmenopausal women 30. These results guide postmenopausal women to adjust their diet, strengthen exercises, and reduce the risk factors of breast cancer that can be controlled artificially.

Breast cancer is age‐dependent, and it is widely accepted that young women tend to present with a greater tumor size that is more advanced and with poorer prognostic characteristics 31, 32. We found that premenopausal women presented with a greater tumor size (more than 2 cm) than postmenopausal women in our study. The fact might imply there are also more aggressive breast cancers in Chinese premenopausal patients than in postmenopausal.

We further analyzed the difference in the pathological features between the two groups. The ER and PR status are important indicators to guide endocrine therapy in breast cancer. It is also an important factor affecting the prognosis of breast cancer 33, 34. Wittliff studied the relationship between menopausal status and ER and reported that the positive expression of ER occurred at a rate of 45% in premenopausal women and at a rate of <63% in postmenopausal patients 35. Anderson reported that ER‐positive rates rose continuously irrespective of menopause 36. However, we found the opposite. That is, the positive expression of ER occurred at a rate of 59% in premenopausal women and was higher than 55% in postmenopausal patients. We also found that postmenopausal patients had higher proportions of positive HER2 expression and positive P53 expression. Scholars have reported a negative correlation between the positive expression of ER and positive HER2 status 37, 38. We obtained similar results. Those results are similar to that in another article from the same database 32. Additionally, higher proportion of (ER+/PR+) in premenopausal patients and higher proportion of (ER−/PR−) in postmenopausal patients suggested that the distribution of hormone receptors in western Chinese women is different from that in foreign countries. The reasons for the difference in the expression of hormone receptor may be as follows: (1) the proportion of premenopausal and postmenopausal women with breast cancer in China is opposite of that in foreign countries 15; (2) the stimulation of different human populations and the external environment affect the expression of hormone receptor; and (3) pregnancy and childbirth lead to the fluctuation of estrogen and progesterone levels, which affect the expression of hormone receptor. The relationship between menopause and ER and PR remains controversial, and we need more large‐scale studies to clarify their relationship.

Regarding treatment options, we compared the following five aspects between the two groups: surgery, chemotherapy, radiotherapy, endocrine therapy, and anti‐HER2 therapy. First, we found that 94% of all patients underwent surgery. Regarding the choice of surgical approach, a higher proportion of premenopausal patients underwent advanced operation methods, such as breast‐conserving surgery and breast reconstruction. A possible explanation was that younger patients have a greater desire to keep their original breasts and shapes and are more accepting of advanced operation methods compared to postmenopausal patients 39. Second, we found that there are more premenopausal patients receiving chemotherapy. Chemotherapy is one of the most commonly used and most effective methods among adjuvant therapies for treating breast cancer. Premenopausal patients had a smaller average age, higher malignancy, and risk of recurrence of the tumors 32, and it was reported that chemotherapy can significantly reduce the risk of relapse of high malignant breast cancer 34, 40, so they could benefit more from chemotherapy. The discovery of more aggressive cancers found in premenopausal women is the fact that leads to more chemotherapy in this population. Third, we found that a higher proportion of premenopausal patients receiving radiotherapy, as radiotherapy is necessary after breast‐conserving surgery, and that the proportion of premenopausal patients receiving breast‐conserving surgery were higher. Additionally, adjuvant radiotherapy after surgery significantly reduces the local recurrence rate and increases the overall survival rate 41. Similarly, the proportion of premenopausal patients receiving endocrine therapy was significantly higher than that of postmenopausal patients. The increased use of endocrine therapy may be due to the fact that endocrine therapy is suitable for hormone receptor‐positive breast cancer and significantly reduces the recurrence rate 42, and the proportion of (ER+/PR+) in premenopausal patients was significantly higher. Finally, this study showed that very few patients with a positive HER2 status accepted anti‐HER2 therapy. The possible reason may be that the HER2 testing condition is deficient in local areas. Additionally, anti‐HER2 therapy is not included in health care, indicating that it is a costly burden in western China.

### Limitations

Our study had some limitations. First, all patients included were from nine provinces of western China; thus, the results may not be generalizable to all women in China. Second, data regarding some characteristics, such as HER2 status, BMI, breastfeeding history, tumor location, P53 status, and Ki67 status, were missing, which may have underpowered the study. Another limitation is that we failed to follow up the patients. As a result, we cannot analyze the relationship between prognosis and clinicopathologic features.

## Conclusion

In this study, we found that the fall in the fertility rate, early menarche age, married, and less breastfeeding might have increased the possibility of premenopausal breast cancer. Significant differences exist in the tumor size, hormone receptor state, HER2 expression, epidemiological features, and treatment modes between premenopausal and postmenopausal female breast cancer patients in western China. The difference in breast cancer onset period remains to be investigated in the further studies.

## Ethical Approval

All procedures performed in studies involving human participants were in accordance with the ethical standards of the institutional and/or national research committee and with the 1964 Helsinki declaration and its later amendments or comparable ethical standards.

## Conflict of Interest

The authors have no disclosures.

## References

[1] Siegel, R. L. , K. D. Miller , S. A. Fedewa , D. J. Ahnen , R. G. Meester , A. Barzi , et al. 2017 Colorectal cancer statistics, 2017. CA Cancer J. Clin. 67:177–193. 28248415 10.3322/caac.21395

[2] Chen, W. , R. Zheng , P. D. Baade , S. Zhang , H. Zeng , F. Bray , et al. 2016 Cancer statistics in China, 2015. CA Cancer J. Clin. 66:115–132. 26808342 10.3322/caac.21338

[3] Anderson, W. F. , P. S. Rosenberg , A. Prat , C. M. Perou , and M. E. Sherman . 2014 How many etiological subtypes of breast cancer: two, three, four, or more? J. Natl Cancer Inst. 106:296–297. 10.1093/jnci/dju165 PMC4148600 25118203

[4] De, W. F. , E. A. Baanders‐Vanhalewijn , and J. Huizinga . 1964 The bimodal age distribution of patients with mammary carcinoma; evidence for the existence of 2 types of human breast cancer. Cancer 17:141–151. 14123673 10.1002/1097-0142(196402)17:2<141::aid-cncr2820170202>3.0.co;2-z

[5] Waard, F. D. 1979 Premenopausal and postmenopausal breast cancer: one disease or two? J. Natl Cancer Inst. 63:549–552. 381750 10.1093/jnci/63.3.549

[6] Fan, L. , Y. Zheng , K. D. Yu , G. Y. Liu , J. Wu , J. S. Lu , et al. 2009 Breast cancer in a transitional society over 18 years: trends and present status in Shanghai, China. Breast Cancer Res. Treat. 117:409–416. 19153831 10.1007/s10549-008-0303-z

[7] Fan, L. , K. Strasser‐Weippl , J. J. Li , J. St Louis , D. M. Finkelstein , K. D. Yu , et al. 2014 Breast cancer in China. Lancet Oncol. 15:e279–e289. 24872111 10.1016/S1470-2045(13)70567-9

[8] Yasui, Y. , and J. D. Potter . 1999 The shape of age‐incidence curves of female breast cancer by hormone‐receptor status. Cancer Causes Control 10:431–437. 10530614 10.1023/a:1008970121595

[9] Tarone, R. E. , and K. C. Chu . 2002 The greater impact of menopause on ER− than ER+ breast cancer incidence: a possible explanation (United States). Cancer Causes Control 13:7–14. 11899120 10.1023/a:1013960609008

[10] Morabia, A. , and M. C. Costanza . 1998 International variability in ages at menarche, first livebirth, and menopause. Am. J. Epidemiol. 148:1195–1205. 9867266 10.1093/oxfordjournals.aje.a009609

[11] Morabia, A. , and P. Flandre . 1992 Misclassification bias related to definition of menopausal status in case‐control studies of breast cancer. Int. J. Epidemiol. 21:222–228. 1428473 10.1093/ije/21.2.222

[12] Elston, C. W. , and I. O. Ellis . 1991 Pathological prognostic factors in breast cancer. I. The value of histological grade in breast cancer: experience from a large study with long‐term follow‐up. Histopathology 19:403–410. 1757079 10.1111/j.1365-2559.1991.tb00229.x

[13] Singletary, S. E. , and J. L. Connolly . 2006 Breast cancer staging: working with the sixth edition of the AJCC Cancer Staging Manual. CA Cancer J. Clin. 56:37–47. 16449185 10.3322/canjclin.56.1.37

[14] Li, J. , B. N. Zhang , J. H. Fan , Y. Pang , P. Zhang , S. L. Wang , et al. 2011 A nation‐wide multicenter 10‐year (1999–2008) retrospective clinical epidemiological study of female breast cancer in China. BMC Cancer 11:364. 21859480 10.1186/1471-2407-11-364 PMC3178543

[15] Althuis, M. D. , J. M. Dozier , W. F. Anderson , S. S. Devesa , and L. A. Brinton . 2005 Global trends in breast cancer incidence and mortality 1973–1997. Int. J. Epidemiol. 34:405–412. 15737977 10.1093/ije/dyh414

[16] Gao, Y. T. , X. O. Shu , Q. Dai , J. D. Potter , L. A. Brinton , W. Wen , et al. 2000 Association of menstrual and reproductive factors with breast cancer risk: results from the Shanghai breast cancer study. Int. J. Epidemiol. 87:295–300. 10.1002/1097-0215(20000715)87:2<295::aid-ijc23>3.0.co;2-7 10861490

[17] Bao, P. P. , X. O. Shu , Y. T. Gao , Y. Zheng , H. Cai , S. L. Deming , et al. 2011 Association of hormone‐related characteristics and breast cancer risk by estrogen receptor/progesterone receptor status in the Shanghai breast cancer study. Am. J. Epidemiol. 174:661–671. 21768404 10.1093/aje/kwr145 PMC3166707

[18] Ali, P. , M. B. A. Fakhree , H. Shahryar , H. Monireh , and D. Amir . 2011 Hormone receptor status in breast cancer and its relation to age and other prognostic factors. Breast Cancer 5:87–92. 21695095 10.4137/BCBCR.S7199 PMC3117624

[19] Lin, C. H. , P. Y. Chuang , C. J. Chiang , Y. S. Lu , A. L. Cheng , W. H. Kuo , et al. 2014 Distinct clinicopathological features and prognosis of emerging young‐female breast cancer in an East Asian country: a nationwide cancer registry‐based study. Oncologist. 19:583–591. 24807917 10.1634/theoncologist.2014-0047 PMC4041679

[20] Linos, E. , D. Spanos , B. A. Rosner , K. Linos , T. Hesketh , J. D. Qu , et al. 2008 Effects of reproductive and demographic changes on breast cancer incidence in China: a modeling analysis. J. Natl Cancer Inst. 100:1352–1360. 18812552 10.1093/jnci/djn305 PMC2556703

[21] Wong, I. O. , B. J. Cowling , C. M. Schooling , and G. M. Leung . 2007 Age‐period‐cohort projections of breast cancer incidence in a rapidly transitioning Chinese population. Int. J. Cancer 121:1556–1563. 17437268 10.1002/ijc.22731

[22] Ademuyiwa, F. O. , and O. I. Olopade . 2003 Racial differences in genetic factors associated with breast cancer. Cancer Metastasis Rev. 22:47–53. 12716036 10.1023/a:1022259901319

[23] Byrne, C. , J. L. Connolly , G. A. Colditz , and S. J. Schnitt . 2015 Biopsy confirmed benign breast disease, postmenopausal use of exogenous female hormones, and breast carcinoma risk. Cancer 89:2046–2052. 10.1002/1097-0142(20001115)89:10<2046::aid-cncr3>3.0.co;2-f 11066044

[24] Chen, W. Y. , S. E. Hankinson , S. J. Schnitt , B. A. Rosner , M. D. Holmes , and G. A. Colditz . 2004 Association of hormone replacement therapy to estrogen and progesterone receptor status in invasive breast carcinoma. Cancer 101:1490–1500. 15378477 10.1002/cncr.20499

[25] Hamajima, N. , K. Hirose , K. Tajima , T. Rohan , E. E. Calle , H. C. Jr , et al. 2002 Alcohol, tobacco and breast cancer – collaborative reanalysis of individual data from 53 epidemiological studies, including 58 515 women with breast cancer and 95 067 women without the disease. Br. J. Cancer 87:1234–1245. 12439712 10.1038/sj.bjc.6600596 PMC2562507

[26] http://www.wpro.who.int/topics/adolescent_health/china_fs.pdf, 2014.

[27] Li, L. , J. Ji , J. B. Wang , M. Niyazi , Y. L. Qiao , and P. Boffetta . 2012 Attributable causes of breast cancer and ovarian cancer in China: reproductive factors, oral contraceptives and hormone replacement therapy. Chin. J. Cancer Res. 24:9–17. 23359757 10.1007/s11670-012-0009-y PMC3555252

[28] Goss, P. E. , K. Strasser‐Weippl , B. L. Lee‐Bychkovsky , L. Fan , J. Li , Y. Chavarri‐Guerra , et al. 2014 Challenges to effective cancer control in China, India, and Russia. Lancet Oncol. 15:489–538. 24731404 10.1016/S1470-2045(14)70029-4

[29] Varghese, C. , and H. R. Shin . 2014 Strengthening cancer control in China. Lancet Oncol. 15:484–485. 24731401 10.1016/S1470-2045(14)70056-7

[30] Renehan, A. G. , M. Tyson , M. Egger , R. F. Heller , and M. Zwahlen . 2008 Body‐mass index and incidence of cancer: a systematic review and meta‐analysis of prospective observational studies. Lancet 371:569–578. 18280327 10.1016/S0140-6736(08)60269-X

[31] Anders, C. K. , D. S. Hsu , G. Broadwater , C. R. Acharya , J. A. Foekens , Y. Zhang , et al. 2008 Young age at diagnosis correlates with worse prognosis and defines a subset of breast cancers with shared patterns of gene expression. J. Clin. Oncol. 26:3324–3330. 18612148 10.1200/JCO.2007.14.2471

[32] Ke, W. , R. Yu , H. Li , Z. Ke , J. Jiang , T. Zou , et al. 2016 Comparison of clinicopathological features and treatments between young (≤40 years) and older (>40 years) female breast cancer patients in west China: a retrospective, epidemiological, multicenter, case only study. PLoS ONE 11:e0152312. 27031236 10.1371/journal.pone.0152312 PMC4816508

[33] Gao, J. D. , J. Wang , X. L. Feng , Y. X. Zhong , and X. Wang . 2009 Characterization of hormone receptor status in 5758 Chinese females with breast cancer. Chin. J. Oncol. 31:683–686. 20021865

[34] Early Breast Cancer Trialists’ Collaborative Group . 2005 Effects of chemotherapy and hormonal therapy for early breast cancer on recurrence and 15‐year survival: an overview of the randomised trials. Lancet 365:1687–1717. 15894097 10.1016/S0140-6736(05)66544-0

[35] Wittliff, J. L. 1984 Steroid‐Hormone receptors in breast cancer. Cancer 53:630–643. 6692266 10.1002/1097-0142(19840201)53:3+<630::aid-cncr2820531308>3.0.co;2-3

[36] Anderson, W. F. , P. S. Rosenberg , I. Menashe , A. Mitani , and R. M. Pfeiffer . 2008 Age‐related crossover in breast cancer incidence rates between black and white ethnic groups. J. Natl Cancer Inst. 100:1804–1814. 19066264 10.1093/jnci/djn411 PMC2639327

[37] Lamy, P. J. , T. Verjat , M. Paye , A. C. Servanton , J. Grenier , P. Leissner , et al. 2006 NASBA: a novel approach to assess hormonal receptors and ERBB2 status in breast cancer. Clin. Chem. Lab. Med. 44:3–12. 16375577 10.1515/CCLM.2006.002

[38] Lee, A. , W. C. Park , H. W. Yim , M. A. Lee , G. Park , and K. Y. Lee . 2007 Expression of c‐erbB2, cyclin D1 and estrogen receptor and their clinical implications in the invasive ductal carcinoma of the breast. Jpn. J. Clin. Oncol. 37:708–714. 17940078 10.1093/jjco/hym082

[39] Huang, N. , M. Liu , J. Chen , B. Yang , J. Xue , C. Quan , et al. 2016 Surgical management of breast cancer in China: a 15‐year single‐center retrospective study of 18502 patients. Medicine 95:e4201. 27828839 10.1097/MD.0000000000004201 PMC5106045

[40] Early Breast Cancer Trialists’ Collaborative Group . 1998 Polychemotherapy for early breast cancer: an overview of the randomised trials. Lancet 352:930–942. 9752815

[41] Clarke, M. , R. Collins , S. Darby , C. Davies , P. Elphinstone , V. Evans , et al. 2007 Effects of radiotherapy and of differences in the extent of surgery for early breast cancer on local recurrence and 15‐year survival: an overview of the randomised trials. Lancet 366:2087–2106. 10.1016/S0140-6736(05)67887-7 16360786

[42] Hackshaw, A. , M. Baum , T. Fornander , B. Nordenskjold , A. Nicolucci , K. Monson , et al. 2009 Long‐term effectiveness of adjuvant goserelin in premenopausal women with early breast cancer. J. Natl Cancer Inst. 101:341–349. 19244174 10.1093/jnci/djn498 PMC2650713

